# The MAPme Project: Implementing a College-Based Study of Substance Use and Mental Wellness

**DOI:** 10.3390/ijerph23081084

**Published:** 2026-08-20

**Authors:** Whitney L. Barfield-Steward, Yijin Xiang, Dalora Najera, Jenipher Ober-Oluoch, Chelsie Benca-Bachman, Hope Derricott, Rameez Syed, Justine W. Welsh, Rohan H. C. Palmer

**Affiliations:** 1 Behavioral Genetics of Addiction Laboratory, Department of Psychology, Emory University, Atlanta, GA 30322, USA; whitney.barfield@emory.edu (W.L.B.-S.); yijinxia@usc.edu (Y.X.); dnajera@emory.edu (D.N.); jaober@emory.edu (J.O.-O.); chelsie_benca-bachman@brown.edu (C.B.-B.); hope.derricott@emory.edu (H.D.); rameez.ali.syed@emory.edu (R.S.); 2 Department of Psychiatry and Human Behavior, Brown University, Providence, RI 02906, USA; 3 Department of Psychiatry and Behavioral Sciences, School of Medicine, Emory University, Atlanta, GA 30329, USA; justine.welsh@emory.edu

**Keywords:** alcohol, community health, health education, mental health, other drugs

## Abstract

**Highlights:**

**Public health relevance—How does this work relate to a public health issue?**

- The stress of transitioning to and from adulthood may have a negative impact on college student performance and health outcomes.
- While in college, students face high rates of mental health disorders (anxiety, depression, suicidal ideation), substance use disorder, severe sleep deprivation, and poor nutrition.

**Public health significance—Why is this work of significance to public health?**

- The MAPme Project establishes a community-engaged college health and wellness surveillance system that monitors mental health and substance trends and informs targeted interventions for colleges/universities.
- Student Engagement, Education, and Empowerment: Leveraging the uniqueness of college/universities as a place of learning can mitigate risks, improve academic success, and address health disparities and access, serving as a microcosm for community health.

**Public health implications—What are the key implications or messages for practitioners, policy makers and/or researchers in public health?**

- MAPme transforms colleges and universities into Learning Health Systems, where data continuously informs practice and campus policy—enabling accountability and responsiveness.
- Access to scalable, longitudinal, real-world evidence will enable more scalable, high-impact science.

**Abstract:**

Background: Transitional age youth aged 18–25 years are more likely to engage in risky behaviors, displaying significantly higher alcohol and illicit drug use levels than adolescents and older adults. College students are particularly vulnerable at this critical life juncture as transitioning to adulthood may impact emotional health and well-being, contributing to anxiety, depression, and feelings of hopelessness, all of which are linked to increased risk of substance use and disorders. Objective: This study characterizes the design and status of the MAPme Project, a prospective project centered on substance use and emotional wellness during and beyond college. Herein, we describe the 2018–2024 pilot cohort that was followed during the first two years of college, remotely during the COVID-19 pandemic, and upon the return to campus. We also describe our latest 2025+ cohort with ongoing data collection. Participants: The 2018–2024 cohort comprised 301 college freshmen who completed online assessments at baseline (Wave 1). The 2025+ cohort has currently enrolled 618 students and counting. Methods: We provide descriptive information on both cohorts, along with preliminary associations of pre- and early collegiate substance use outcomes, psychopathology, sleep patterns, and personality traits. Results: Approximately 64% of first-year students reported use of a substance in the 90 days leading up to college in 2018. Alcohol and cannabis were the most prevalent substances used in both cohorts with males consuming more alcohol than females. Females reported greater levels of daily stressors and mood symptoms. Other health and personality characteristics and observed associations between psychosocial risk and protective factors are described. Prior studies demonstrate the ability to test research hypotheses in the social, health, and clinical areas of psychology with robust statistical power. Conclusions: Substance use and emotional well-being vary considerably upon entry into college/university and at any particular moment in time. The MAPme Project encourages the engagement of students to address challenges associated with lack of awareness, inclusivity, and acceptance of scientific research. Overcoming barriers of inclusivity and empowering the community is key to the successful adoption and implementation of campus health and community-oriented research programs and systematically enhancing research training.

## 1. Introduction

Young adults aged 18–25 have the highest rates of alcohol and illicit drug use, with college students particularly vulnerable to heavy drinking and substance use disorders (SUDs) [1,2,3,4]. Nearly half of college students meet criteria for cannabis or alcohol use disorders during their first three years of college [5]. These behaviors are associated with significant health, academic, social, and legal consequences [6,7].

The development of SUDs reflects a complex interplay of genetic, developmental, psychological, environmental, and sociocultural influences [8]. The transition to college introduces new freedoms, social networks, academic demands, and stressors that can increase vulnerability to anxiety, depression, and substance misuse [9,10]. Although many students reduce their substance use after graduation, those with greater underlying susceptibility are more likely to progress to persistent SUDs [11,12]. Understanding how biological, psychological, and environmental factors interact over time is therefore essential for identifying individuals at greatest risk.

Despite decades of research, most college substance use studies have been cross-sectional, conducted at single institutions, and have disproportionately sampled students of European ancestry [13,14,15,16]. These limitations reduce the generalizability of findings and hinder understanding of how cultural, environmental, and socioeconomic factors contribute to SUD development. Large, diverse, longitudinal studies are needed to characterize risk trajectories and identify opportunities for early intervention.

The current Project Report describes our ongoing effort to address these gaps by describing current and past cohorts of the Behavioral Genetics of Addiction Laboratory’s MAPme Project that was launched in 2018. The MAPme Project is a multi-site longitudinal study examining substance use and mental health across the college years and into early adulthood. MAPme integrates demographic, behavioral, psychological, sociocultural, and genetic data while employing targeted recruitment strategies to ensure diverse representation. The project’s long-term goal is to establish a community-centered biorepository that supports interdisciplinary research, expands participation among historically underrepresented populations, and advances evidence-based approaches to promoting student mental health and preventing substance use disorders. Although the initial pilot cohort concluded in 2024, it provided valuable insights into conducting large-scale, multi-site research and informed the development of the ongoing 2025+ cohort. Here, we describe the MAPme conceptual framework, community engagement strategies, and dissemination efforts.

### The MAPme Conceptual Model

**Figure 1:**
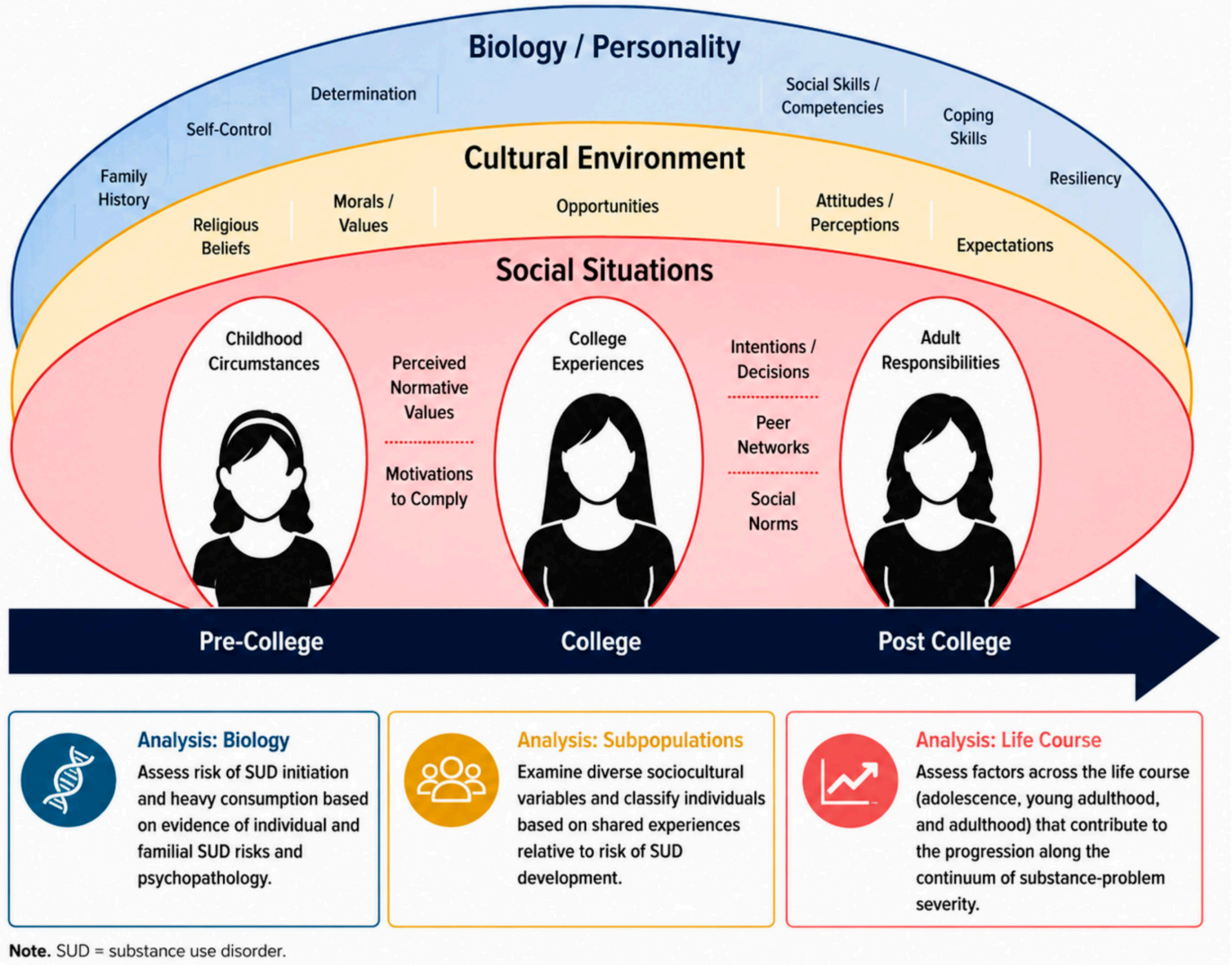
The MAPme Conceptual Model. Based on the TTI, MAPme explores both proximal and distal factors associated with substance use development including biology, cultural environments, and social situations.

MAPme is guided by the Theory of Triadic Influence, which recognizes that substance use arises from interacting biological, psychological, interpersonal, and sociocultural influences [17]. The longitudinal framework follows students from before college through the years after graduation, capturing stable characteristics (e.g., genetics, sex, personality, race/ethnicity) alongside changing environmental influences, including peer networks, campus climate, social norms, and life experiences. This design enables examination of both risk and protective factors, allowing researchers to identify pathways leading to substance use initiation, escalation, recovery, or resilience across young adulthood. Our longitudinal model, presented in Figure 1, illustrates many influencers related to the development of SUD at different time points across young adulthood, particularly life during and immediately after college (ages 18–26). Each stage of our model considers those generally static characteristics of an individual, including biological factors such as sex, genetic predisposition, personality, and race/ethnicity. It also incorporates other foundational cultural, sociodemographic, intrapersonal (inherited) factors and personal experiences that may influence their behavior at baseline. Next, we capture data on external environmental influences at several points along the student journey—before entering college, the college years, and the immediately following years. The objective is to assess the impact of the microenvironment, which includes social norms and peer pressures that may affect health behaviors, along with the broader macroenvironment, such as the campus community that may perpetuate use through normative values or beliefs, campus climate, and expectations.

## 2. Methods

### 2.1. Program Assessments

The 2018–2024 MAPme cohort used a longitudinal design from fall 2018 to summer 2024 to collect a wide range of information on 303 individuals to provide insight into the factors that affect substance use development and emotional wellness in young adults. Starting in 2025, MAPme converted to a cohort study that tracks individuals (undergraduate and graduate) in college. Each semester, college-attending students, 18 and older, who comprehend written English, provide responses to survey instruments via multiple recruitment systems (i.e., classrooms, Student Online Neuroscience Assessment (SONA), and advertising flyers). To capture this information, we have modified and leveraged several questionnaires in the domains of cognition, psychopathology, substance use, demographics, personality, health behaviors, and sociocultural data. Behavioral data are collected using the Research Electronic Data Capture (REDCap) (available online: https://project-redcap.org/ (accessed on 4 August 2025)) web-based application tool, a user-friendly interface to collect and store data. Below we discuss baseline assessments that were captured at Wave 1. A complete list of the questionnaires included in subsequent waves can be found in Table 1.

All study procedures were reviewed and approved by the Emory University Institutional Review Board (IRB) and at each participating institution. Electronic informed consent was obtained from all participants prior to enrollment, including explicit consent for long-term storage of biospecimens and future genetic and genomic analyses. Participant data and biospecimens were de-identified and assigned unique study identifiers to protect confidentiality. Research data were stored on secure, password-protected, encrypted servers with access limited to authorized study personnel, while biospecimens were maintained in a secure biorepository under controlled environmental conditions. All data collection, storage, sharing, and genetic analyses were conducted in accordance with institutional policies, applicable federal regulations, and relevant ethical guidelines.

### 2.2. Participant Recruitment and Program Structure

The 2018–2024 cohort targeted students enrolled as freshmen at both the Emory University and Oxford campuses in the 2018 fall semester. Students were deemed eligible to participate if they were first-time freshmen at the start of recruitment and were 18 years of age or older; freshman status was independently verified using a double-blind approach. In the two weeks leading up to the beginning of the semester, students were actively recruited via two mechanisms. Firstly, study coordinators posted flyers in approved, heavily trafficked sites across the campus, including on-campus freshman dormitories. Secondly, a group of more senior undergraduate student ambassadors, volunteering on the project, hosted activities around campus and virtually to promote the study. They also visited classrooms in person and online to disseminate program flyers and related information. Prospective participants were incentivized by the opportunity to receive a $15 gift card for completion of the study assessments at each wave. Advertising booths were also set up at different locations around campus to promote the study. If recruited at the events hosted by the undergraduate ambassadors, students also had a chance to receive a free MAPme T-shirt, cup, phone wallet, and/or water bottle.

All interested students were provided with a flyer that contained a QR code or website link that allowed them to access the study assessments. Upon visiting the project website, students were immediately presented with an electronic consent form. After completing the consent form, the participants were subsequently led through a series of surveys that focus on risky health behaviors, personality characteristics, emotional wellness, sociodemographic information, substance use, personal experiences, family history, and cognition. After completing the surveys at Wave 1, students were then given the option to visit the BGA laboratory in-person and opt in to provide a saliva sample for DNA extraction (see the Frequently Asked Questions on our website for details on consent, data security, and privacy protections [18]); participants who consented to provide DNA received an additional $5 gift card at the time of compensation. To encourage consented students to continue participating in the subsequent waves, they were re-contacted via email reminders and recruitment materials at tabling events on campus. Participation was also promoted via a raffle draw where participants at each site have a chance to receive a gift card at each wave of assessment. To ensure that we captured pertinent information on students at various time points throughout the year, we created a comprehensive testing schedule to include all essential questionnaires at each wave without overburdening the participants. The pilot cohort completed a total of 10 assessments with no further plans to recontact this group of students beyond their second year post-graduation. Herein, we describe the features of the first year of college of these students. As with any longitudinal study, the 2018–2024 cohort experienced attrition during the over the years with different students returning and leaving the study over time (fall 2019 n = 138; spring 2020 n = 90; fall 2020 n = 117, spring 2021 n = 111, fall 2021 n = 103; spring 2022 n = 80, summer 2023 n = 30; summer 2024 n = 49), which spanned the COVID-19 pandemic. The occurrence of COVID-19 prompted us to include survey instruments related to COVID-related stress (Table 1).

**Table 1:** Selected MAPme assessment domains, constructs, administration/scoring details, references, and linked source files.

Domain	Assessment/Measure	Purpose/Construct	Administration and Scoring Details	Reference(s)
Sociodemographic Information	Sociodemographic questionnaire	Collects age, race/ethnicity, sex, marital status, annual family income, family structure, and hometown population.	Descriptive questionnaire; no scoring algorithm specified in the supplied description.	MAPme sociodemographic questionnaire
Substance Use	Alcohol Use Disorders Identification Test (AUDIT)	Identifies risk of harmful alcohol use using 10 items.	Three items assess quantity/frequency, four assess alcohol-related problems, and three assess alcohol dependence. AUDIT-C contains 3 items scored 0–12; positive cutoffs are ≥4 for men and ≥3 for women in the supplied description. AUDIT-P ranks risk relative to problematic behaviors over the past year.	[19,20]
Substance Use	Alcohol, Smoking, and Substance Involvement Screening Test (ASSIST)	Screens for problematic alcohol, smoking, and substance involvement.	Eight questions classify low, moderate, and high risk. In the supplied description, scores of 0–3 indicate low risk, 4–26 moderate risk, and 27+ high risk.	[21]
Substance Use	TRACER	Measures internal experience of alcohol addiction irrespective of consumption levels, that is related to craving, thoughts about drinking, emotionality, and loss of control; adapted versions are also used for cannabis and tobacco/nicotine.	Twelve questions, with each item scored from 0 to 4.	[22]
Substance Use	Compulsive alcohol use (adapted for cannabis, tobacco, nicotine)	Measures compulsive use among individuals who use alcohol; adapted versions are used for cannabis and tobacco/nicotine.	Composed of questions adapted from the Obsessive Compulsive Drinking Scale, Alcohol Craving Questionnaire, Impaired Control scale, and Alcohol Use Questionnaire.	[23,24,25]
Substance Use	E-Cigarette Use	Assesses electronic cigarette consumption and related perceptions.	Eight questions on lifetime e-cigarette use, frequency of use, previous daily use, perceived harm, preferred flavor, nicotine content, and primary reasons for use.	[20]
Substance Use	WHO ASSIST V3.0	Assesses recent use of alcohol, tobacco products, and other substances.	Eight questions about experiences over the past three months. For endorsed substances, frequency options range from never to daily or almost daily.	[21]
Personality	Big Five Inventory (BFI)	Assesses five common personality dimensions.	Forty-four items measuring openness, conscientiousness, extraversion, agreeableness, and neuroticism.	[26]
Personality	UPPS-P Impulsive Behavior Scale	Assesses impulsive personality traits.	Fifty-nine items covering negative urgency, positive urgency, lack of premeditation, sensation seeking, and lack of perseverance.	[27]
Psychopathology	Generalized Anxiety Disorder-7 (GAD-7)	Assesses anxiety symptoms.	Participants report symptoms over the past two weeks on a 0–3 scale from not at all to nearly every day.	[28]
Psychopathology	Perceived Stress Scale (PSS)	Assesses self-reported perception of stress.	Fourteen items ask participants to rate how they deal with situations or external triggers over the last month on a 0–4 scale from never to very often.	[29]
Psychopathology	Patient Health Questionnaire-9 (PHQ-9)	Assesses severity of depressive symptoms.	Nine DSM-IV symptom items scored from 0 to 3, from not at all to nearly every day. The supplied description notes that it can be used as a pre-screen to identify at-risk individuals.	[30]
Sleep Quality	Pittsburgh Sleep Quality Index (PSQI)/Sleep Quality	Measures sleep quality and distinguishes poor from good sleep quality.	PSQI items capture seven areas: subjective sleep quality, sleep latency, sleep duration, habitual sleep efficiency, sleep disturbances, use of sleeping medications, and daytime dysfunction over the last month.	[31]

MAPme continues with our 2025+ cohort that targets undergraduate and graduate students enrolled at any year of college/university in the United States of America. We primarily reach students by identifying and contacting faculty and administrators at colleges and universities each semester to establish partnerships and obtain IRB approval to recruit from their site. In doing so, the survey is accessible to students through a myriad of classrooms and via flyers on their campus. Providing DNA is no longer a requirement for participation in MAPme, and students who choose to participate can opt for compensation in the form of assignment/research participation credit in their course or to freely contribute to science. Students can participate multiple times during a semester and throughout college; each event is treated as a separate enrollment and timestamped. All identifying information in our data is removed to protect participants.

### 2.3. Engaging, Educating, and Empowering a Community Using Research

In addition to the defined scientific objectives of MAPme, the program also serves as an educational research hub at the University—engaging undergraduate and graduate students to participate as student ambassadors and empowering them to be thoughtful research advocates. While college students represent a unique subset of the 18–25-year-old demographic that is at risk for using substances, many regard them as an opportunistic population because of a willingness to meet the expectations of faculty/investigators who hold a position of authority at an institution [28]. To overcome this potential confounder we made study participation voluntary, provided monetary compensation, and allowed individuals the opportunity to opt out at any time during or after data collection. As such, MAPme incurs the same challenges as random and stratified sampling. To keep participants aware and engaged with the study, we actively create and offer programming (e.g., speaker seminars) within the broader community under study, regardless of whether the student has participated previously. In doing so, the entire campus benefits from access to education and research resources. For instance, we employ graduate student RAs who gain hands-on experience by working closely with doctoral researchers to co-lead and support various aspects of the program. Graduate students lead four critical components of the program, including the (1) Quantitative Core, which assists with describing the data and performing advanced quantitative data analyses, (2) REDCAP Assessments Core, which develops online survey and oversees participant compensation, (3) the Education & Outreach Core that is responsible for carrying out community engagement and outreach activities, and (4) the Media Core that develops content for our websites and social media platforms to disseminate activities and findings to MAPme participants and the general public. Each graduate student is assigned 1–3 undergraduate student ambassador(s), who work together to develop a comprehensive strategic plan to complete essential program objectives every semester. Undergraduate students develop practical skills in hypothesis generation, data analysis, computational methodologies, recruitment, and health outreach. The MAPme data are also utilized in two research-focused psychology courses for undergraduates (PSYCH 223, PSYCH 480) and graduates (PSYCH 720P). Students in these courses can generate and test hypotheses while building up their knowledge of the biological and sociocultural bases that impact college substance use.

All MAPme trainees and ambassadors are encouraged to develop and participate in the programmatic outreach and engagement efforts. Thus far, we have implemented a bi-annual seminar series to spotlight prominent community-based behavioral and genetic studies and scientists advancing the research. For example, principal investigators with the All of Us research program [32] and Spit for Science [33] have led informational discussions with students on campus to inform them of their research efforts and ask clarifying questions. Additionally, we host bi-monthly tabling events on campus to provide students with information about MAPme-sponsored social engagements such as study breaks, general wellness activities, and informative discussions/forums.

### 2.4. Data Analyses

Below we report basic demographic information and statistical data of select variables of interest for the pilot and 2025+ cohorts. Total and group sample means, the prevalence for specific outcomes of interest, and summary scores for select behavioral assessments were performed. We estimated measures of dispersion (i.e., standard deviation) for these descriptive statistics where appropriate. Tetrachoric correlations were used to determine the inter-correlation of each of the substance use variables. In addition, we highlighted recently published papers led by undergraduate and graduate trainees who tested hypotheses using these data in a laboratory [34,35] and classroom [36] setting.

## 3. Results

### 3.1. Demographics Information on the MAPme Cohorts

In total, the pilot (2018–2024) cohort recruited 305 first-year Emory University students in Wave 1 of the study. This group comprised 6.4% of incoming freshmen (N = 1964, n = 1431 at the Atlanta campus, and n = 533 at Oxford) during a record year of enrollment at the university. The 2018–2024 cohort comprised 44.5% White (non-Hispanic), 14.5% Asian, 10% Black, 7% Hispanic or Latino, 3% Two or More Races, 0.1% American Indian or Alaska Native, and 0.08% Native Hawaiian or Other Pacific Islanders. Of those that reported on sex (N = 301), 70% (n = 212) were female. The majority (57%; n = 171) of the MAPme participants come from suburban environments (57%; n = 171), followed by those from urban communities (38%; n = 114), then rural environments with the least representation (5%; N = 15). A total of 272 participants (89%) consented to provide a saliva sample for DNA extraction.

To date, the 2025+ cohort has recruited 618 students (mean age = 20.64, SD = 2.99, age range = 18 to 37) across seven US-based college campuses. While data collection is ongoing each semester, as of fall 2026, 28%% (n = 171) were male and 72% (n = 443) were female. All participants consented to the study under their own site IRB. No DNA samples were collected from this cohort. As of fall 2025, the 2025+ cohort comprises 48.4% White (non-Hispanic), 26.7% Asian, 13.8% Black, 14.1% Hispanic or Latino, 3.6% Two or More Races, 1.5% American Indian or Alaska Native, and 0.0% Native Hawaiian or Other Pacific Islanders.

### 3.2. Patterns of Substance Use in the MAPme Cohorts

Determination of drug use was based on self-report data obtained using the Alcohol, Smoking and Substance Involvement Screening Test (ASSIST) [21]. Among first years in the 2018–2024 cohort approximately 64% (N = 193) of individuals reported using any substance (i.e., alcohol, tobacco, cannabis, cocaine, etc.) one or more times in their lifetime (see Table 1). There were no significant gender differences in substance initiation. In this cohort, alcohol was the most prevalent substance used (66% [N = 189]) followed by cannabis (28% [N = 83] and cigarettes (26% [N = 77]) at the start of college. The rest of the substances were reported at low rates; in descending order, sedatives were reported at 12%, amphetamines at 9%, hallucinogens at 8%, cocaine at 4%, opioids at 3% and inhalants at 2% (Table 2). Among the students in the 2025+ cohort of MAPme, which comprises a wider age range of students at different years in college, alcohol was the most prevalent substance used in their lifetime (69%), followed by cannabis (36%), and cigarettes (23%). Opioids were the least endorsed substance (2%) across campuses. No gender differences were observed. A notable change between the two cohorts, is that almost a decade later, the prevalence rates are similar, with a slight increase of 1–2%.

Of growing interest in the field of substance use has been the phenomenon of polysubstance use; to tap into this behavior, we show the correlations of various substances reported by in Table 3. As expected, “ever use” of alcohol was most closely associated with the use of cannabis and cigarette smoking. This observation has remained consistent after the COVID-19 pandemic in the 2025+ cohort.

Table 4 summarizes student involvement with substances. Based on the ASSIST scale, among students who endorsed alcohol use, most were at low risk of problems. While both men and women similarly report a low risk of alcohol-related problems, there was a significantly higher risk among men in both cohorts. The remaining substances were reported at too low rates on the ASSIST for meaningful statistical differentiation. According to the AUDIT scale, males and females had similar total scores, but males consumed more on average. Males also consumed and/or binged alcohol more frequently, as reported by the AUDIT-C (2018–2024 cohort: μ_males_ = 4.42; μ_females_ = 3.19; t-value = −2.66, *p* = 0.010; 2025+ cohort: μ_males_ = 4.04; μ_females_ = 3.06; t-value = 3.66, *p* < 0.001). Regarding compulsive drinking, on average, student craving for alcohol fell within the “minimal social drinking” range for first-year students (2018–2024 cohort-μ = 5.92), whereas individuals using tobacco/nicotine and cannabis exceed the threshold of 7 or higher indicating that these individuals were dependent. By comparison, mean levels of alcohol, tobacco/nicotine, and cannabis compulsive use in the 2025+ cohort indicated greater dependence on average.

### 3.3. Stress and Psychopathology

When examining participants’ degree of stress levels using the Perceived Stress Scale, both male and female students reported moderate stress levels; however, females reported higher stress levels than males in both cohorts (Table 5). Depression symptoms, as examined via the PHSQ-9, were reported only minimally by male students; however, females reported significantly higher (mild) levels. These patterns are consistent with anxiety as well, with males reporting only minimal levels of anxiety, and females, significantly higher, mild levels via the GAD assessment [28].

### 3.4. Sleep Patterns

Table 6 describes patterns and quality of sleep. On average, students in the 2018–2024 cohort reported μ = 7.95 (standard deviation (SD) = 2.17) hours of sleep on weekdays (μ_males_ = 7.87 (2.32)); μ_females_ = 8.01 (2.05)), with slightly more sleep reported on weekends (8.62 (3.00); (μ_males_ = 8.33 (2.95); μ_females_ = 8.79 (2.97)). Majority (75.6%) report having good (64.7%) to very good (10.9%) sleep quality, with the remaining reporting poor (19.8%) to very poor (4.6%) quality sleep. The patterns of sleep were similar in the 2025+ cohort, which saw an average weekday sleep duration of 7.02 h (SD = 2.00) versus 8.08 (SD = 2.00) hours on the weekend. There were no sex differences in sleep behaviors observed.

### 3.5. Impulsivity and Personality

Table 7 describes the personality facets of each cohort using the UPPS impulsivity scale [27] and the BFI [26] for the 2018–2024 and 2025+ cohorts. When assessing the UPPS scale results, we found significantly higher scores for both lack of premeditation subscale (μ_males_ = 1.92; μ_females_ = 1.81; *p*-value = 0.039) and sensation seeking subscale (μ_males_ = 2.93; μ_females_ = 2.64; *p*-value < 0.001) among males, indicating more impulsive behavior through the two pathways. This observation was also observed in the 2025+ cohort (premeditation μ_males_ = 3.00; μ_females_ = 3.13; *p*-value < 0.001; sensation seeking μ_males_ = 2.85; μ_females_ = 2.62; *p*-value < 0.001).

On the BFI assessment, males had a higher average score in extraversion than females (μ_males_ = 3.42; μ_females_ = 3.09; *p*-value = 0.004), indicating that males were more likely to be extroverted. On the contrary, males had a lower average score in Neuroticism (Negative Emotionality) than females (μ_males_ = 2.85; μ_females_ = 3.31; *p*-value < 0.001), indicating that males tend to have more stable emotions. Gender differences in the 2025+ cohort did not follow the same pattern, possibly due to the wider age range and differences in lived experiences thus far. Among students in the 2025+ cohort, females reported higher levels of conscientiousness μ_males_ = 3.39; μ_females_ = 3.69; *p* < 0.001), neuroticism (μ_males_ = 2.80; μ_females_ = 3.30; *p* < 0.001), and open-mindedness (μ_males_ = 3.52; μ_females_ = 3.64; *p* = 0.029) on average.

### 3.6. Past Empirical Findings

Examinations of the 2018–2024 pilot data have led to three research studies that have informed how temperament, personality, and behavior characteristics of studies relate to their mood, sleep behaviors, and substance use. Using data gathered during the first year of college demonstrated that neurotic tendencies was a direct indicator of both depressed mood and poor sleep quality with evidence to suggest that sleep and mood directly impacted each other over time [34]. In a separate study, focused on behavioral reports at the start of college, ref. [35] compared the associations between personality traits and stress with alcohol use/misuse. Findings demonstrated that associations between daily stressors and alcohol involvement was influenced by a student’s tendency to experience mood swings, anxiety, irritability, and sadness. Specifically, among students who displayed a high number of depression-like neurotic tendencies, experiencing more daily stressors was associated with greater alcohol use/misuse. On the contrary, students who were low on these depression-like neurotic tendencies, used/misused alcohol less often as their level of daily stressor increased. A Brown et al. study [36], as part of a graduate course on the “Behavioral Effects of Drugs”, leveraged MAPme data to examine whether temperament and behavioral characteristics of students mediated familial effects on drug use. The lead authors showed that a family history of internalizing, drinking, and illicit substance use, as well as teen personality and temperament are associated with drinking and alcohol problems, both in the 90 days prior and during college. To date, there have been two studies using data collected over the course of the COVID-19 pandemic. In a multi-site collaboration [37], we examined COVID-19-related stressors impacted alcohol consumption on college students in MAPme and a group of students from an institution in the southwestern United States. We saw that higher levels of COVID-19-related stress, negative urgency, and sensation seeking increased the frequency of alcohol consumption and alcohol use disorder (AUD) symptoms. Moreover [33,38], drinking problems worsened if teens experiencing a COVID-19 stressor were generally more prone to having greater negative urgency.

## 4. Discussion

MAPme is a college-based study established to analyze distal and proximal factors that lead to substance use and related disorders. We have developed a comprehensive set of behavioral, psychological, and cognitive assessments to capture a wide range of information from young adults. To date, students in the pilot and 2025+ cohorts have completed surveys and engaged with student representatives. MAPme participant’s rates of substance use are comparable or slightly lower in all drug categories relative to findings from other cohort studies of entering freshmen or college students as a whole [33,38]. Larger sample sizes and further examination of our cohort’s socioeconomic background, cultural dynamics, and family history are needed to begin to explain some of the reasons behind these use rates. When examining the inter-correlations between alcohol and the other substances, we found that alcohol initiation was most correlated with marijuana and cigarettes initiation. These findings are consistent with the literature, as these are the most comorbid substances in this population since the decline in smoking in the early 2000s [39].

Early studies using the 2018–2024 cohort showed that students differ in a multiplicity of dimensions in the time leading up to and during the first year of college. When examining alcohol consumption, assessing the AUDIT-C, most students were at low risk of harmful consumption. Males, however, had higher AUDIT-Consumption scores. Similarly for depression, stress, and anxiety rates in students, the level of problem severity were generally mild; however, there were significant sex differences noted in each category—women reporting higher levels in all three. Our findings support the literature, as many studies suggest that the female sex may be more vulnerable to chronic stress, resulting in increased mood deficits relative to their male counterparts. Therefore, it will be imperative to understand these differences in pathophysiology over time, particularly as it relates to substance use development in this population.

Most of the freshman students in the 2018–2024 cohort surveyed reported having good to very good sleep duration throughout the week, with the highest rates of sleep occurring on the weekend. It will be important however, to examine how these students’ sleep behavior changes each year, as studies suggest that as a student’s classification increases from freshman, sophomore, junior, to senior, their reported sleep duration decreases significantly [40]. The decrease in sleep quality has significant implications, as poor sleep is associated with drug use, smoking, and substance abuse. By comparison, levels in the 2025+ cohort appear slightly lower for a heterogenous group of students, who on average, endorsed having good quality sleep and 7–8 h across the weekdays and weekend, indicating that almost a decade later there has been a slight downward shift across cohorts.

We also note several sex differences in personality facets among our cohorts, which were not always consistent, warranting further study. In both the 2018–2024 and 2025+ cohorts, males reported more impulsive behaviors—specifically, lack of premeditation and sensation seeking which is consistent with the past literature [41,42]. Considering that impulsivity is an important risk factor for problematic substance use and substance use disorders later in life, it will be important to see if these differences in use begin to manifest later in the college experience in males [43]. On the contrary, females, reported higher levels of neuroticism than their male counterparts. Individuals with higher levels of neuroticism have been found to be more likely to smoke cigarettes and misuse alcohol over the life course, so gaining a greater understanding of these differences will be key in understanding how personality predicts substance-use behaviors in young adulthood [44]. A notably difference between the two cohorts in terms of personality traits is that males exhibited much greater levels of externalizing in the 2018–2024 cohort, which was expected given their younger age on average compared to the 2025+ cohort [45]. From the earliest studies of MAPme it is evident that several theoretical explanations for individual differences in personality, stress, emotionality, and substance use are supported. Support for the genetic/biological basis of substance use implies that these transmissible effects are not behavior specific and may manifest in the form of emotional, temperament, or substance use behaviors. This is aligned with recent findings on the hierarchical taxonomy of psychopathology which suggest that genetic effect on mental health behaviors mirror the same correlational structure observed among their behavioral counterparts with evidence for higher-order dimensions [46,47]. Relatedly, although support for the self-medication hypothesis is mixed, Brown et al.’s cross-sectional study suggests that a challenge to testing this theory may stem from substance use behaviors being driven by multiple liabilities that may express themselves in similar forms due to unknown reasons not explicitly modeled in prior research [48].

### 4.1. Programmatic Observations

In addition to surveying students, we are taking additional strides to build rapport with the undergraduate community. As such, we have developed several programmatic efforts that go above and beyond basic recruitment. These activities include annual seminars, tabling events on campus, a psychology course that allows students to review MAPme data, a website [49], and social media accounts to disseminate information, and summative data sharing with campus administrators to facilitate compliance with federal guidelines for campus drinking policies. These combined efforts enable the team to interact with other students, faculty, and staff, who may or may not participate in the study, educate them, and obtain direct feedback about the program. The MAPme Project also empowers graduate and undergraduate students as research assistants and ambassadors by providing them with the opportunity to get hands-on research experience for consecutive years. Evidence suggests that students who become involved in on-campus research experience numerous direct benefits. In many cases, they are exposed to broader research experiences that can be explored in the classroom and gain an avenue to learn more about scientific career paths early on to aid decision-making post-college. Ultimately, these interactions increase the likelihood of a student going on to pursue a scientific career [40].

### 4.2. Considerations and Limitations

We note several areas of planning and implementation that may be of interest to others interested in developing similar studies on their college campus. First, because the original MAPme 2018–2024 cohort employed a longitudinal cohort design, we limited recruitment at baseline to the first six weeks of the semester. A consequence of limiting recruitment to a small window was the ascertainment of a smaller sample size. However, it is difficult to ascertain how much larger the cohort might have been with an extended period. Spit for Science, by comparison, allowed students to join at any point throughout college. Still, capturing 7% of the student body is statistically well above the margin for bias [50], but we cannot exclude the possibility that our study pool comprises individuals with a strong tendency to engage in research, Second, as the MAPme cohort continues to be assessed, we have observed varying levels of participation across subsequent waves. The variable retention rates are consistent with most longitudinal studies but underscore the importance of continuous participant engagement, allowing students to join at later time points. To overcome these challenges, future cohorts of MAPme (described below) adopt a pseudo-longitudinal design. Third, many of the assessments are limited to self-reports, which may be prone to biases and false reporting. However, we attempt to mitigate this concern by using multiple assessments and screening for erroneous lifetime responses across assessment waves (when the data are available). Lastly, while both cohorts represent the demographics of their campuses, because recruitment was never designed to test differences between demographic groups, larger samples are needed to draw robust conclusions about group differences. While preliminary results are informative, continued efforts to broaden participation across all demographic groups will improve generalizability and support research advances that benefit a wider range of communities.

### 4.3. Ongoing Efforts

In the spring of 2025, the MAPme Study relaunched data collection of its newest cohort across multiple colleges and universities in the southeastern United States and active recruitment on other sites that is ongoing. In this new 2025+ cohort we focus on building community partnerships with university deans, campus administrators, faculty and student organizations. We also work closely with campus faculty and staff to first obtain a site IRB, and then work to coordinate recruitment efforts, and facilitate new educational events on campus. A major strength of MAPme is our willingness to share resources to enhance student preparedness and wellness through greater understanding and education. For example, in the past, we held focus groups to better understand the barriers and facilitators to participation in genetically informed research on substance use/misuse and health behaviors in college-aged youth. Similarly, faculty at our Emory site can opt to apply to include de-identified MAPme data as an educational resource in their courses.

**Figure 2:**
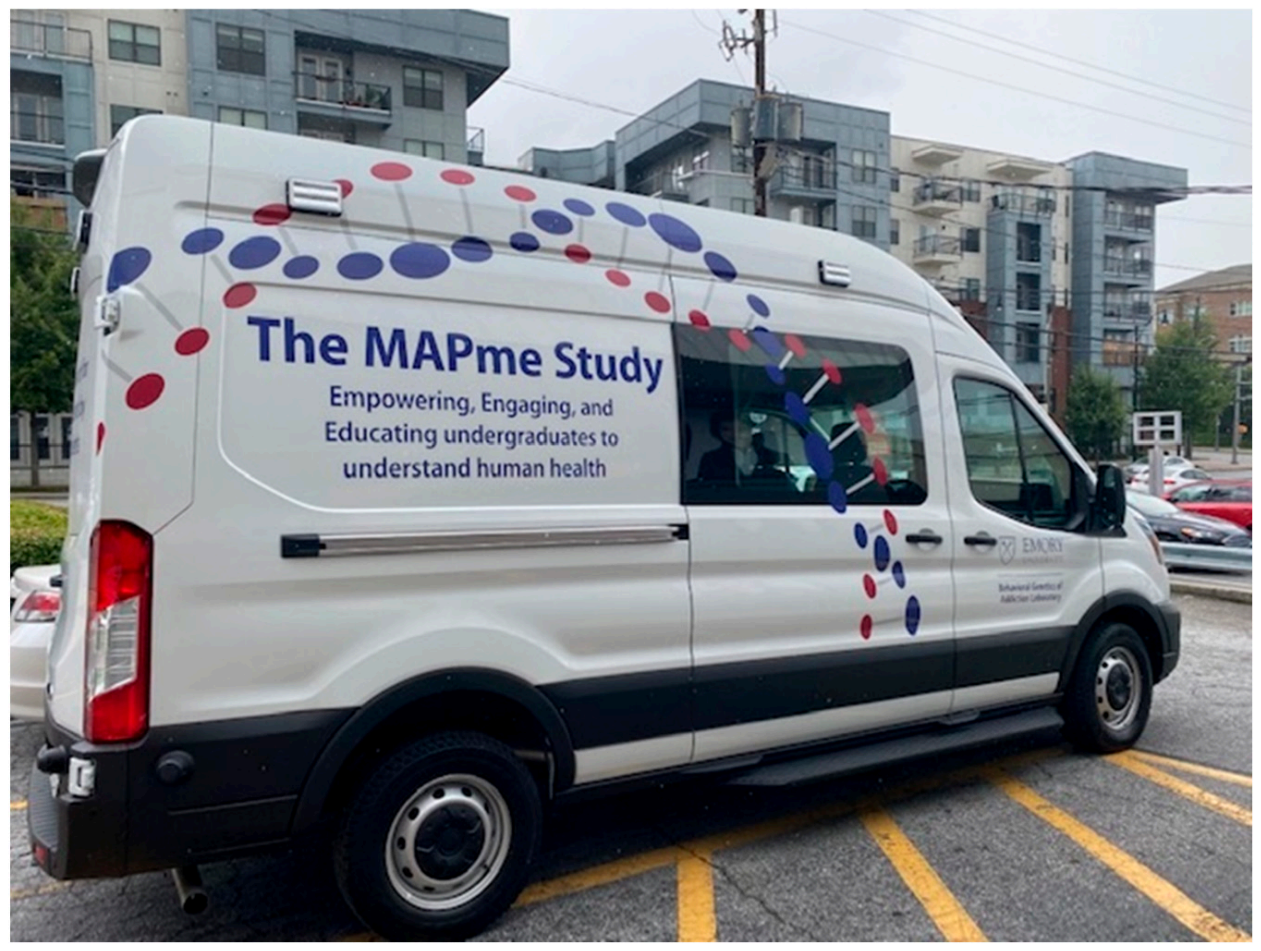
The MAPme Van. Photograph depicting the MAPme Van used to transport recruitment and engagement tools, as well as members of the research team to events.

Additionally, to make participation as seamless as possible for students and further diversify data collection methods to include observational and performance measures, we built a mobile research vehicle to meet participants where they live and go to school. The research van (Figure 2) serves as a base of operations for student engagement and disseminating recruitment and educational materials. The van can transport a team of three researchers and comes equipped with iPads for data collection, a Wi-Fi hotspot, and refrigerated cooling to facilitate the in-person collection of saliva kits. These additional tools facilitated greater access to students and student engagement and helped to stem attrition due to the COVID-19 pandemic, which saw elevated rates of emotional distresss [51], uncertainty [52], and drinking among college students [53,54]. Our student engagement efforts also highlighted the need to shift from a passive longitudinal strategy to a more active pseudo-longitudinal design that (1) meets students where they are on campus (i.e., the classroom), (2) emphasizes the importance of engagement in research through research credits in courses, (3) allows for more flexible participation (i.e., across classes), (4) compensates students in multiple ways that are most salient to them (participant-centered reports, research credit) and (5) builds partnerships with faculty (consortium membership and data sharing). All these observations of our program’s effectiveness underscored the MAPme recruitment design and relaunch in spring 2025. By placing greater emphasis on community partnerships with faculty and university administrators, and by meeting students where they are—in the classroom, by partnering with existing SONA infrastructures and using MAPme’s topics as a medium to bring research papers directly into the classroom and faculty lab using MAPme’s de-identified dataset, we obtained the same recruitment numbers in a single semester and doubled the numbers by the second semester, demonstrating our ability to reach students and assess them wherever they are in the United States.

Details on how to become a part of MAPme are available on the MAPme website [55]. Interested parties/faculty can complete our form [56] to request to be added to our multi-campus panel.

To bring MAPme to your college or university, please visit the Project Requirement page on our website [55] or complete the Faculty Information/Collaboration form [56]. Participants can engage with MAPme through various activities, including data sharing, reporting results, joint publications, and more. By filling out the collaboration form, you can help us align our efforts with your specific interests. Faculty and/or administrators can bring MAPme into their classrooms by filling out the Faculty Information/Collaboration form [56], which collects essential details about the course where the survey will be presented. Local IRB approval may be required.

We will carry out further analysis on the data from the subsequent waves of the survey to investigate how the substance use varied among our participants. Additionally, we will be reporting the changes in the pattern of substance use upon graduation and leaving college.

## 5. Conclusions

Forming a multi-site community-driven research and education program is challenging; still, the benefits (i.e., cultivating a more educated and engaged at-risk population, increased research training opportunities for undergraduate and graduate students) outweigh the risks. Prospectively engaging college students transcends opportunistic sampling. Our efforts will provide much-needed answers to trends in mental health and wellness nationwide, as well as emerging challenges in tackling barriers to inclusion of at-risk individuals and diverse student representation at all levels of the research process.

## Figures and Tables

**Table 2:** Ever use statistics of the MAPme study population, stratified by gender.

Baseline Characteristic	Total (N = 301)		Male (N = 89)		Female (N = 212)		t ^a/b^	*p*
	M ^a^/N ^b^	SD ^a^/% ^b^	M ^a^/N ^b^	SD ^a^/% ^b^	M ^a^/N ^b^	SD ^a^/% ^b^		
MAPME cohort 2018–2024								
Age (yrs) ^a^	18.58	0.39	18.65	0.38	18.55	0.39	−2.19	0.03
Emory Main Campus	193	0.64	62.00	0.70	131.00	0.62	1.36	0.24
Ever use—Any substance	194	0.65	52.00	0.59	142.00	0.67	1.49	0.22
Ever use—Alcohol	189	0.63	50.00	0.57	139.00	0.66	1.82	0.18
Ever use—Cigarettes	77	0.26	22.00	0.25	55.00	0.26	0.01	0.94
Ever use—Cannabis	83	0.28	26.00	0.30	57.00	0.27	0.11	0.74
Ever use—Cocaine	4	0.01	2.00	0.02	2.00	0.01	2.41	0.58
Ever use—Amphetamine	9	0.03	3.00	0.03	6.00	0.03	1.20	0.73
Ever use—Inhalants	2	0.01	0.00	0.00	2.00	0.01	0.00	1.00
Ever use—Sedatives	12	0.04	4.00	0.04	8.00	0.04	1.20	0.75
Ever use—Hallucinogens	8	0.03	4.00	0.04	4.00	0.02	2.44	0.24
Ever use—Opioids	3	0.01	2.00	0.02	1.00	0.00	4.82	0.21
MAPME cohort 2025+								
	Total (N = 611)		Male (N = 169)		Female (N = 442)		t ^a/b^	*p*
	M ^a^/N ^b^	SD ^a^/% ^b^	M ^a^/N ^b^	SD ^a^/% ^b^	M ^a^/N ^b^	SD ^a^/% ^b^		
Age (yrs) ^a^	21	2.99	20.73	2.74	20.61	3.08	0.49	0.63
Ever use—Any substance	419	0.69	110.00	0.65	309.00	0.70	1.29	0.26
Ever use—Alcohol	408	0.67	108.00	0.64	300.00	0.68	0.70	0.40
Ever use—Cigarettes	141	0.23	44.00	0.26	97.00	0.22	0.93	0.33
Ever use—Cannabis	219	0.36	65.00	0.39	154.00	0.35	0.55	0.46
Ever use—Cocaine	32	0.05	9.00	0.05	23.00	0.05	0.00	1.00
Ever use—Amphetamine	29	0.05	8.00	0.05	21.00	0.05	0.00	1.00
Ever use—Inhalants	21	0.03	7.00	0.04	14.00	0.03	0.12	0.73
Ever use—Sedatives	30	0.05	5.00	0.03	25.00	0.06	1.37	0.24
Ever use—Hallucinogens	50	0.08	16.00	0.10	34.00	0.08	0.30	0.58
Ever use—Opioids	12	0.02	4.00	0.02	8.00	0.02	4.82	0.75

Note. The total number of participants did not sum up to 303 as two participants put “prefer not to answer” to the gender question. ^a^ Continuous variables were displayed as mean (SD). Student’s *t*-test was applied for continuous variables. ^b^ Categorical variables were displayed as N (%). The Chi-square or Fisher’s exact test (for cells having counts less than 5) was applied for categorical variables.

**Table 3:** Correlations for ever use variables.

Variable	1. Ever Use—Alcohol	2. Ever Use—Cigarettes ^a^	3. Ever Use—Cannabis ^a^	4. Ever Use—Other Substance ^b^
MAPme 2018–2024 (N = 305)				
1. Ever use—alcohol ^a^	1.00			
2. Ever use—cigarettes ^a^	0.41 **	1.00		
3. Ever use—cannabis ^a^	0.44 **	0.60 **	1.00	
4. Ever use—Other substance ^b^	0.19	0.25	0.31	1.00
MAPme 2025+ (N = 618)				
1. Ever use—alcohol ^a^	1.00			
2. Ever use—cigarettes ^a^	0.74 **	1.00		
3. Ever use—cannabis ^a^	0.84 **	0.81 **	1.00	
4. Ever use—Other substance ^b^	0.47 **	0.67 **	0.69 **	1.00

Note. Tetrachoric correlations were applied to assess the inter-correlation between substance use variables. ^a^ All variables were ever use over a lifetime. ^b^ Other substances included Cocaine, Amphetamine, Inhalants, Sedatives, Hallucinogens, and Opioids. ** *p* < 0.01.

**Table 4:** Levels of Involvement in Substance Use Behaviors in MAPme-Pilot Cohort at Baseline.

Behavior	Overall (Mean/SD) and Range	By Gender			
		Males	Females	t-Value	*p*-Value
MAPme 2018–2024					
Assist Total Score					
Alcohol	5.47 (4.36)	6.78 (4.40)	4.99 (4.26)	−2.35	0.021
Tobacco	5.03 (5.77)	5.78 (6.65)	4.71 (5.39)	−0.6	0.552
Cannabis	6.27 (5.94)	7.60 (7.99)	5.70 (4.78)	−0.99	0.331
AUDIT					
AUDIT Total Score	4.81 (4.26)	5.68 (4.14)	4.54 (4.29)	−1.52	0.134
AUDIT-C Score	3.50 (2.38)	4.42 (2.75)	3.19 (2.16)	−2.66	0.01
AUDIT-P Score	0.97 (2.05)	0.81 (1.77)	1.03 (2.15)	0.67	0.507
OCDS Total Score					
Alcohol	5.92 (4.17)	5.27 (2.87)	6.36 (4.88)	0.859	0.396
Tobacco	13.20 (14.52)	13.67 (17.62)	12.50 (14.85)	−0.08	0.942
Cannabis	10.11 (6.50)	13.29 (6.87)	8.09 (5.65)	−1.67	0.123
MAPme 2025+					
Assist Total Score					
Alcohol	4.83 (4.60)	5.21 (5.28)	4.69 (4.33)	0.92	0.357
Tobacco	5.92 (7.59)	5.33 (6.26)	6.19 (8.14)	−0.69	0.491
Cannabis	4.88 (6.61)	5.27 (6.92)	4.72 (6.49)	0.56	0.577
AUDIT					
AUDIT Total Score	4.67 (3.79)	5.41 (4.08)	4.39 (3.65)	2.14	0.034
AUDIT-C Score	3.33 (2.09)	4.04 (2.31)	3.06 (1.95)	3.66	<0.001
AUDIT-P Score	1.34 (2.19)	1.37 (2.28)	1.33 (2.17)	0.16	0.871
OCDS Total Score					
Alcohol	16.00 (4.16)	16.50 (4.43)	15.83 (4.05)	1.38	0.169
Tobacco	19.82 (9.80)	19.40 (8.32)	20.01 (10.43)	−0.38	0.708
Cannabis	18.12 (6.98)	18.86 (7.64)	17.82 (6.69)	0.97	0.335

**Table 5:** Psychopathology in MAPme-pilot cohort at baseline.

Behavior	Overall	By Gender			
		Males	Females	t-Value	*p*-Value
MAPme 2018–2024					
Depressed Mood					
PSS Total Score	25.88 (5.39)	24.81 (5.54)	26.28 (5.29)	2.08	0.038
PHQ-9 Score	5.24 (5.67)	4.06 (5.15)	5.69 (5.82)	2.37	0.019
Anxiety					
GAD Total Score	5.45 (5.14)	4.23 (4.89)	5.94 (5.17)	2.69	0.008
MAPme 2025+					
Depressed Mood					
PSS Total Score	26.19 (7.21)	24.18 (7.73)	26.96 (6.85)	−4.11	<0.001
PHQ-9 Score	5.87 (5.82)	4.40 (5.23)	6.43 (5.94)	−4.12	<0.001
Anxiety					
GAD Total Score	6.36 (5.56)	4.16 (4.65)	7.20 (5.65)	−6.79	<0.001

**Table 6:** Sleep Patterns in MAPme-pilot cohort at baseline.

Behavior	Overall	By Gender			
		Males	Females	t-Value	*p*-Value
MAPme 2018–2024					
Sleep (Hours per day)					
Sleep Duration for a Typical Weekday	7.95 (2.17)	7.87 (2.32)	8.01 (2.05)	1.23	0.221
Sleep Duration for a Typical Weekend	8.62 (3.00)	8.33 (2.95)	8.79 (2.97)	1.44	0.696
Sleep Quality (n)					
Very Good	33 (10.9%)	12 (13.5%)	21 (9.9%)		
Good	196 (64.7%)	55 (61.8%)	139 (65.6%)		
Poor	60 (19.8%)	19 (21.3%)	41 (19.3%)		
Very Poor	14 (4.6%)	3 (3.4%)	11 (5.2%)		
MAPme 2025+					
Sleep (Hours per day)	Overall	by gender			
		Males	Females	t-value	*p*-value
Sleep Duration for a Typical Weekday	7.02 (2.00)	7.10 (1.54)	6.99 (2.16)	0.70	0.484
Sleep Duration for a Typical Weekend	8.08 (2.00)	8.14 (2.07)	8.06 (1.97)	0.41	0.685
Sleep Quality (n)				2.57	0.462
Very Good	52 (8.5%)	15 (8.9%)	37 (8.4%)		
Good	373 (61.0%)	110 (65.1%)	263 (59.5%)		
Poor	147 (24.1%)	34 (20.1%)	113 (25.6%)		
Very Poor	35 (5.7%)	8 (4.7%)	27 (6.1%)		

**Table 7:** Personality scales in MAPme pilot cohort at baseline.

Behavior	Overall	By Gender			
		Males	Females	t-Value	*p*-Value
MAPme 2018–2024					
UPPS-P					
Premeditation	1.84 (0.43)	1.92 (0.44)	1.81 (0.43)	−2.05	0.039
Urgency	2.21 (0.59)	2.15 (0.63)	2.23 (0.57)	1.03	0.281
Sensation seeking	2.73 (0.59)	2.93 (0.57)	2.64 (0.58)	−3.94	<0.001
Perseverance	1.94 (0.50)	1.97 (0.54)	1.92 (0.48)	−0.65	0.495
BFI					
Extraversion	3.18 (0.90)	3.42 (0.97)	3.09 (0.86)	−2.78	0.004
Agreeableness	3.86 (0.67)	3.76 (0.67)	3.91 (0.67)	1.79	0.074
Conscientiousness	3.62 (0.63)	3.51 (0.69)	3.67 (0.60)	1.88	0.062
Negative Emotionality	3.17 (0.80)	2.85 (0.83)	3.31 (0.76)	4.45	<0.001
Open-Mindedness	3.66 (0.57)	3.63 (0.56)	3.67 (0.57)	0.583	0.564
MAPme 2025+					
	Overall	By gender			
UPPS-P		Males	Females	t-value	*p*-value
Premeditation	3.09 (0.37)	3.00 (0.38)	3.13 (0.36)	−3.87	<0.001
Urgency	2.29 (0.54)	2.34 (0.54)	2.28 (0.55)	1.29	0.199
Sensation seeking	2.68 (0.56)	2.85 (0.47)	2.62 (0.57)	5.22	<0.001
Perseverance	2.95 (0.34)	2.89 (0.33)	2.97 (0.34)	−2.66	0.008
BFI					
Extraversion	3.13 (0.84)	3.09 (0.81)	3.15 (0.85)	−0.79	0.43
Agreeableness	3.90 (0.62)	3.86 (0.63)	3.91 (0.61)	−0.83	0.406
Conscientiousness	3.61 (0.67)	3.39 (0.71)	3.69 (0.64)	−4.88	<0.001
Negative Emotionality	3.16 (0.80)	2.80 (0.78)	3.30 (0.76)	−7.09	<0.001
Open-Mindedness	3.61 (0.59)	3.52 (0.55)	3.64 (0.60)	−2.19	0.029

## Data Availability

Data are available upon request to the senior author as they are already a part of a repository. Please visit: MAPme—Behavioral Genetics of Addiction Laboratory or contact rohan.palmer@emory.edu.

## References

[1] Tucker J.S., Rodriguez A., Davis J.P., Klein D.J., D’Amico E.J. (2021). Simultaneous trajectories of alcohol and cannabis use from adolescence to emerging adulthood: Associations with role transitions and functional outcomes. Psychol. Addict. Behav..

[2] Claros E., Sharma M. (2021). The relationship between emotional intelligence and abuse of alcohol, marijuana, and tobacco among college students. J. Alcohol Drug Educ..

[3] Bingham C.R., Shope J.T., Tang X. (2005). Drinking behavior from high school to young adulthood: Differences by college education. Alcohol Clin. Exp. Res..

[4] Patrick M.E., Terry-McElrath Y.M., Evans-Polce R.J., Schulenberg J.E. (2020). Negative alcohol-related consequences experienced by young adults in the past 12 months: Differences by college attendance, living situation, binge drinking, and sex. Addict. Behav..

[5] Caldeira K.M., Kasperski S.J., Sharma E., Vincent K.B., O’Grady K.E., Wish E.D., Arria A.M. (2009). College students rarely seek help despite serious substance use problems. J. Subst. Abus. Treat..

[6] Jackson K.M., Sokolovsky A.W., Gunn R.L., White H.R. (2020). Consequences of alcohol and marijuana use among college students: Prevalence rates and attributions to substance-specific versus simultaneous use. Psychol. Addict. Behav..

[7] Stamates A.L., Yang M., Lau-Barraco C. (2023). Validation of the Brief Young Adult Alcohol Consequences Questionnaire among student and nonstudent young adults. Exp. Clin. Psychopharmacol..

[8] Gerring Z.F., Thorp J.G., Treur J.L., Verweij K.J.H., Derks E.M. (2024). The genetic landscape of substance use disorders. Mol. Psychiatry.

[9] Schulenberg J.E., Sameroff A.J., Cicchetti D. (2004). The transition to adulthood as a critical juncture in the course of psychopathology and mental health. Dev. Psychopathol..

[10] Borsari B., Murphy J.G., Barnett N.P. (2007). Predictors of alcohol use during the first year of college: Implications for prevention. Addict. Behav..

[11] Kroshus E., Hawrilenko M., Browning A. (2021). Stress, self-compassion, and well-being during the transition to college. Soc. Sci. Med..

[12] Walukevich-Dienst K., Calhoun B.H., Fairlie A.M., Cadigan J.M., Patrick M.E., Lee C.M. (2023). Using substances to cope with social anxiety: Associations with use and consequences in daily life. Psychol. Addict. Behav..

[13] D’Amico E.J., Tucker J.S., Shih R.A., Miles J.N. (2014). Does diversity matter? The need for longitudinal research on adolescent alcohol and drug use trajectories. Subst. Use Misuse.

[14] Sirugo G., Williams S.M., Tishkoff S.A. (2019). The Missing Diversity in Human Genetic Studies. Cell.

[15] Haardorfer R., Windle M., Fairman R.T., Berg C.J. (2021). Longitudinal changes in alcohol use and binge-drinking among young-adult college students: Analyses of predictors across system levels. Addict. Behav..

[16] Morales A.M., Jones S.A., Kliamovich D., Harman G., Nagel B.J. (2020). Identifying Early Risk Factors for Addiction Later in Life: A Review of Prospective Longitudinal Studies. Curr. Addict. Rep..

[17] Flay B.R., Petraitis J. (1994). The Theory of Triadic Influence: A New Theory of Health Behavior with Implications for Preventive Interventions. Advances in Medical Sociology.

[18] Behavioral Genetics of Addiction Laboratory. Frequently Asked Questions. https://scholarblogs.emory.edu/mapme/faqs/.

[19] Babor T., de la Fuente J., Saunders J., Grant M. (1989). AUDIT, The Alcohol Use Disorders Identification Test: Guidelines for Use in Primary Health Care.

[20] Fleming M.F., Barry K.L., MacDonald R. (1991). The alcohol use disorders identification test (AUDIT) in a college sample. Int. J. Addict..

[21] WHO ASSIST Working Group (2002). The Alcohol, Smoking and Substance Involvement Screening Test (ASSIST): Development, reliability and feasibility. Addiction.

[22] Palmer R.H. (2026). Transdiagnostic Research-Based Assessment of Compulsive Ethanol Risk (TRACER©).

[23] Anton R.F., Moak D.H., Latham P. (1995). The Obsessive Compulsive Drinking Scale: A self-rated instrument for the quantification of thoughts about alcohol and drinking behavior. Alcohol. Clin. Exp. Res..

[24] Raabe A., Grusser S.M., Wessa M., Podschus J., Flor H. (2005). The assessment of craving: Psychometric properties, factor structure and a revised version of the Alcohol Craving Questionnaire (ACQ). Addiction.

[25] Heather N., Tebbutt J.S., Mattick R.P., Zamir R. (1993). Development of a scale for measuring impaired control over alcohol consumption: A preliminary report. J. Stud. Alcohol..

[26] John O.P., Srivastava S. (1999). The Big-Five trait taxonomy: History, measurement, and theoretical perspectives. Handbook of Personality: Theory and Research.

[27] Lynam D.R., Smith G.T., Whiteside S.P., Cyders M.A. (2006). The UPPS-P: Assessing Five Personality Pathways to Impulsive Behavior.

[28] Spitzer R.L., Kroenke K., Williams J.B., Lowe B. (2006). A brief measure for assessing generalized anxiety disorder: The GAD-7. Arch. Intern. Med..

[29] Cohen S., Spacapan S., Oskamp S. (1988). Perceived stress in a probability sample of the United States. The Social Psychology of Health.

[30] Kroenke K., Spitzer R.L., Williams J.B. (2001). The PHQ-9: Validity of a brief depression severity measure. J. Gen. Intern. Med..

[31] Buysse D.J., Reynolds C.F., Monk T.H., Berman S.R., Kupfer D.J. (1989). The Pittsburgh Sleep Quality Index: A new instrument for psychiatric practice and research. Psychiatry Res..

[32] Sankar P.L., Parker L.S. (2017). The Precision Medicine Initiative’s All of Us Research Program: An agenda for research on its ethical, legal, and social issues. Genet. Med..

[33] Dick D.M., Nasim A., Edwards A.C., Salvatore J.E., Cho S.B., Adkins A., Meyers J., Yan J., Cooke M., Clifford J. (2014). Spit for Science: Launching a longitudinal study of genetic and environmental influences on substance use and emotional health at a large US university. Front. Genet..

[34] Catherman C., Cassidy S., Benca-Bachman C.E., Barber J.M., Palmer R.H.C. (2023). Associations between neuroticism, subjective sleep quality, and depressive symptoms across the first year of college. J. Am. Coll. Health.

[35] Martin K.P., Benca-Bachman C.E., Palmer R.H.C. (2021). Risk for alcohol use/misuse among entering college students: The role of personality and stress. Addict. Behav. Rep..

[36] Brown A.L., España R.A., Benca-Bachman C.E., Welsh J.W., Palmer R.H. (2020). Adolescent Behavioral Characteristics Mediate Familial Effects on Alcohol Use and Problems in College-Bound Students. Subst. Abus. Res. Treat..

[37] Mcnamara I.A., Jaume-Feliciosi N., Jamil B., Benca-Bachman C.E., Palmer R.H.C., Su J. (2026). COVID-19 Related Stress, Impulsivity, and Alcohol Use Outcomes among College Students. J. Dual Diagn. Res. Pract. Subst. Abus. Comorbidity.

[38] Bono R.S., Barnes A.J., Dick D.M., Kendler K.S. (2017). Drinking, Cigarette Smoking, and Employment Among American College Freshmen at a Four-Year University. Subst. Use Misuse.

[39] White J., Walton D., Walker N. (2015). Exploring comorbid use of marijuana, tobacco, and alcohol among 14 to 15-year-olds: Findings from a national survey on adolescent substance use. BMC Public Health.

[40] Raley H., Naber J., Cross S., Perlow M. (2016). The Impact of Duration of Sleep on Academic Performance in University Students. Madridge J. Nurs..

[41] Weafer J., de Wit H. (2014). Sex differences in impulsive action and impulsive choice. Addict. Behav..

[42] Weisberg Y.J., Deyoung C.G., Hirsh J.B. (2011). Gender Differences in Personality across the Ten Aspects of the Big Five. Front. Psychol..

[43] Mitchell M.R., Potenza M.N. (2015). Importance of sex differences in impulse control and addictions. Front. Psychiatry.

[44] Turiano N.A., Whiteman S.D., Hampson S.E., Roberts B.W., Mroczek D.K. (2012). Personality and Substance Use in Midlife: Conscientiousness as a Moderator and the Effects of Trait Change. J. Res. Pers..

[45] Ferguson H.J., Brunsdon V.E.A., Bradford E.E.F. (2021). The developmental trajectories of executive function from adolescence to old age. Sci. Rep..

[46] Kotov R., Krueger R.F., Watson D., Achenbach T.M., Althoff R.R., Bagby R.M., Brown T.A., Carpenter W.T., Caspi A., Clark L.A. (2017). The Hierarchical Taxonomy of Psychopathology (HiTOP): A dimensional alternative to traditional nosologies. J. Abnorm. Psychol..

[47] Waldman I.D., Poore H.E., Luningham J.M., Yang J. (2020). Testing structural models of psychopathology at the genomic level. World Psychiatry.

[48] Neale M.C., Kendler K.S. (1995). Models of comorbidity for multifactorial disorders. Am. J. Hum. Genet..

[49] MAPme. https://scholarblogs.emory.edu/mapme/.

[50] Cochran W.G. (1977). Sampling Techniques.

[51] Zimmermann M., Bledsoe C., Papa A. (2021). Initial impact of the COVID-19 pandemic on college student mental health: A longitudinal examination of risk and protective factors. Psychiatry Res..

[52] Huang A., Liu L., Wang X., Chen J., Liang S., Peng X., Li J., Luo C., Fan F., Zhao J. (2023). Intolerance of uncertainty and anxiety among college students during the re-emergence of COVID-19: Mediation effects of cognitive emotion regulation and moderation effects of family function. J. Affect. Disord..

[53] Jackson K.M., Merrill J.E., Stevens A.K., Hayes K.L., White H.R. (2021). Changes in Alcohol Use and Drinking Context due to the COVID-19 Pandemic: A Multimethod Study of College Student Drinkers. Alcohol. Clin. Exp. Res..

[54] Mohr C.D., Umemoto S.K., Rounds T.W., Bouleh P., Arpin S.N. (2021). Drinking to Cope in the COVID-19 Era: An Investigation Among College Students. J. Stud. Alcohol. Drugs.

[55] Behavioral Genetics of Addiction Laboratory MAPme Project Participant Requirements. https://scholarblogs.emory.edu/mapme/participant-requirements/.

[56] MAPme Collaboration Form. https://forms.gle/puC7T2zS91td3rA98.

